# Maturation of three-dimensional, hiPSC-derived cardiomyocyte spheroids utilizing cyclic, uniaxial stretch and electrical stimulation

**DOI:** 10.1371/journal.pone.0219442

**Published:** 2019-07-05

**Authors:** Wesley LaBarge, Saidulu Mattappally, Ramaswamy Kannappan, Vladimir G. Fast, Daniëlle Pretorius, Joel L. Berry, Jianyi Zhang

**Affiliations:** 1 Department of Biomedical Engineering, The University of Alabama at Birmingham, Birmingham, Alabama, United States of America; 2 School of Medicine, The University of Alabama at Birmingham, Birmingham, Alabama, United States of America; University of Tampere, FINLAND

## Abstract

Functional myocardium derived from human induced pluripotent stem cells (hiPSCs) can be impactful for cardiac disease modeling, drug testing, and the repair of injured myocardium. However, when hiPSCs are differentiated into cardiomyocytes, they do not possess characteristics of mature myocytes which limits their application in these endeavors. We hypothesized that mechanical and electrical stimuli would enhance the maturation of hiPSC-derived cardiomyocyte (hiPSC-CM) spheroids on both a structural and functional level, potentially leading to a better model for drug testing as well as cell therapy. Spheroids were generated with hiPSC-CM. For inducing mechanical stimulation, they were placed in a custom-made device with PDMS channels and exposed to cyclic, uniaxial stretch. Spheroids were electrically stimulated in the C-Pace EP from IONOptix for 7 days. Following the stimulations, the spheroids were then analyzed for cardiomyocyte maturation. Both stimulated groups of spheroids possessed enhanced transcript and protein expressions for key maturation markers, such as cTnI, MLC2v, and MLC2a, along with improved ultrastructure of the hiPSC-CMs in both groups with enhanced Z-band/Z-body formation, fibril alignment, and fiber number. Optical mapping showed that spheroids exposed to electrical stimulation were able to capture signals at increasing rates of pacing up to 4 Hz, which failed in unstimulated spheroids. Our results clearly indicate that a significantly improved myocyte maturation can be achieved by culturing iPSC-CMs as spheroids and exposing them to cyclic, uniaxial stretch and electrical stimulation.

## Introduction

With the discovery of human induced pluripotent stem cells (hiPSCs), which possess the ability to be differentiated into any cell type of any organ system from a patient’s own cells, the potential avenues for regenerative medicine and the discovery of patient-specific treatments grew tremendously [1]. hiPSC-derived cardiomyocytes (hiPSC-CMs) could one day be used in cellular therapy to replace the damaged myocardium so as to prevent heart failure after myocardial infarction (MI) [2, 3]. In recent studies, hiPSC-CMs, endothelial cells, and smooth muscle cells were transplanted into a heart post MI and these cells were shown to aid in both functional and structural improvements in the post MI heart in various animal models [2, 4]. A major issue with the use of hiPSC-CMs in drug discovery, cell therapy, and disease modeling is that these cells are phenotypically immature which do not adequately represent the adult cardiomyocyte, limiting their usability [5].

hiPSC-CMs differ from mature adult myocytes in a few key aspects. hiPSC-CMs exhibit much higher ratio of nuclei/cytoplasmic volume [6]. The calcium handling ability of these cells is reduced compared to adult cells [7]. Immature hiPSC-CMs do express important cardiac gap junctions such as connexin 43 (CX43) and N-cadherin (N-Cad) that are also expressed in adult myocytes. However, the arrangement of these gap junctions in these types of cells display a more circumferential distribution like that in fetal cardiac cells and the expression is significantly lower [8]. Therefore, the effective maturation of hiPSC-CMs is paramount for the successful applications in drug discovery, disease modeling, and therapeutic interventions [9, 10].

Three dimensional hiPSC-CM spheroids present a useful avenue for drug testing as they can be cultured rapidly in large amounts for cell therapy through bioreactor culture as well as in small well plates for high-throughput drug screening. Also, in spheroids, cells interact on a three dimensional level better representing how adult cardiomyocytes interact *in vivo* [11–13]. However, despite their three dimensional architecture, they remain an immature cell type which significantly limits their applications.

We hypothesized that exposing a scaffold-free spheroid model composed of hiPSC-CMs to regimens of chronic electrical stimulation or cyclic, uniaxial stretch for 7 days will enhance the maturation of the cardiomyocytes on both the structural and functional levels.

In the present study, we subjected three-dimensional, hiPSC-CM spheroids to cyclic, uniaxial stretch in a novel structure as well as chronic electrical stimulation. Proof of the effectiveness of the uniaxial stretching system as well as the electrical stimulation setup were first confirmed and the extent of maturation of the spheroids exposed to each regimen was characterized through transcript expression analyses using RT-qPCR, cardiac-specific protein immunolabeling of key maturation markers, and ultrastructural evaluations using transmission electron microscopy (TEM). Functional characterization of conduction velocity, action potential duration, and determination of the minimum cycle length threshold was done through optical mapping.

## Materials and methods

### hiPSC-CM culture

Human cardiac fibroblast derived iPSCs for this project were differentiated into cardiomyocytes as previously reported from a single cell line [14]. Briefly, after hiPSC selection, cardiomyocyte differentiation commenced using a protocol initially utilizing 6 μM CHIR99021 in RPMI-1640 (Gibco) basal medium supplemented with B27 minus insulin (B27-). One day later, fresh RPMI with B27- was added and cultured for two days. Then the process continued with the cells being cultured in RPMI basal medium with B27- and the addition of the Wnt signaling inhibitor 10 μM IWR1 for two days, followed by two additional days of culturing in RPMI supplemented with B27 plus insulin (B27+). Around this time, spontaneously beating hiPSC-CMs appeared and the cells were then further metabolically selected as previously reported. In short, on day 10, the cardiomyocyte differentiation medium was substituted for cardiomyocyte selection medium. This medium consisted of RPMI-1640 medium minus glucose supplemented with 4 mM lactate and B27+. Differentiation medium was added back to the cells for one additional day 48 hours later. On days 13 through 16, this same medium change from differentiation medium to selection medium was repeated. After this time, trypsinization of the monolayers occurred using 0.25% trypsin-EDTA for 5 minutes at 37°C. Trypsinized cells were collected and the process of spheroid formation began.

### hiPSC-CM spheroid culture

The trypsinized hiPSC-CMs were added to medium comprised of RPMI-1640 medium with B27+. These cells were then plated into ultra-low attachment 96 U-well plates (Corning, Product #7007) at concentrations between 2.5×10^3^ to 3×10^5^ cells per well. To generate spheroids with an approximate diameter of 800 μm for this study, a protocol was followed as previously reported from our lab [14]. Briefly, after the addition of the cells to the ultra-low attachment U-wells, cells fused together in the form of spheroids. Spheroids which were approximately 800 μm in diameter formed from the addition of approximately 2.5×10^5^ cells. After 7 days in culture, these cells contracted into compact spheroids. Spheroid culture was done in an incubator at 37°C supplemented with 5% CO_2_. Every 48 hours, fresh medium was added to the spheroids.

### Generation of novel polydimethylsiloxane (PDMS) channels

To act as an elastic substrate to support the cyclic, uniaxial stretching of the hiPSC-CM spheroids, custom molds were made to generate the PDMS channels. The channels were designed to hold spheroids that are ≤ 1 mm in diameter and allow them to attach to the bottom surface for effective stretching (S1A and S1B Fig). PDMS (Dow Corning Sylgard 184 Silicone, Product #2065622) was mixed as prescribed by the company at a 10:1 ratio and added to the mold. These were cured at 75°C for 2 hours in an oven. Fabrication yielded structures with four channels, each 1 mm wide and 8.5 mm in length to accommodate approximately 10 spheroids per channel. The PDMS structures were autoclaved prior to the addition of the spheroids.

### Mechanical stimulation of hiPSC-CM spheroids

After sterilization, the channels were prepared to hold and stretch the spheroids. For attachment of the spheroids to the PDMS surface, Matrigel (Corning Matrigel Matrix, Phenol-Red Free, Product #356231) was mixed with Dulbecco’s Modified Eagle Medium/Nutrient Mixture F12 (DMEM/F12, Thermo Fisher Scientific, Product #11330057) and added to each channel and then placed in an incubator at 37°C supplemented with 5% CO_2_ to solidify. After 1 hour, the Matrigel mixture was removed and spheroids were individually added to the channels in RPMI-1640 media supplemented with B27+ and allowed to settle on the bottom for attachment to the surface overnight. Once attached to the surface, the channel structure with spheroids was secured to a custom designed cyclic, uniaxial stretching device. The moving side of the device was operated using linear actuator motors (TRA12CC Actuators, Newport) that push and release against a spring-compressed bar in a cyclic manner. The movement was controlled using a custom LabVIEW program. With this device, the spheroids were stretched at 10% strain at a frequency of 1 Hz for 7 days (S1C Fig). This particular stretching regimen was determined based on both previously reported findings and stretching limitations of the experimental setup [15]. The media for the device was changed every 48 hours.

### Electrical stimulation of hiPSC-CM spheroids

The hiPSC-CM spheroids were exposed to chronic, electrical stimulation using a monolayer culture-pacing device (C-Pace EP, IONOptix) (S1D Fig). Spheroids were added to 6-well culture plates with media consisting of RPMI-1640 and B27+. The pacing device’s top portion was placed on the culture plate with the stimulating carbon electrodes placed in the well and submerged in the media. The spheroids were stimulated with an electric field of 6.5 V/cm with 5 ms pulses at a frequency of 2 Hz for 7 days. Fresh media was added to the wells every 48 hours and the top portion of the stimulating plate was changed out every 48 hours as well to prevent potential electrolysis byproducts from the electrodes from causing necrosis in the spheroids, as suggested by the company.

### Quantitative RT-PCR analysis

To isolate the total RNA within the spheroids, RNeasy Plus Mini Kits (Qiagen, Product #74134) were utilized. The extracted RNA concentrations were estimated using a spectrophotometer (NanoDrop 2000, Thermo Fisher Scientific, Product #ND-2000). Less than 1 μg of RNA was used per 20 μL reaction using TaqMan Reverse Transcription Reagents (Applied Biosystems, Product #N8080234) to reverse transcribe the RNA with the appropriate primers (S1 Table) for cDNA generation. Quantitative RT-PCR was performed with Fast SYBR Green Master Mix (Applied Biosystems, Product #4385612) on a Realplex^2^ RT-PCR system (Eppendorf). Analysis consisted of using the Ct values to calculate the 2^-ΔCt^ values by using GAPDH transcript levels as the housekeeping gene and calculating the relative expression to the control group.

### SEM imaging

PDMS channels (without Matrigel and with Matrigel) were prepared for imaging by first fixing with 4% paraformaldehyde (PFA) for 15 minutes at room temperature. The PFA was then removed and the specimens were subsequently submerged in liquid nitrogen for approximately 5 seconds. Once removed from the liquid nitrogen, the frozen channels were placed in 50 mL Falcon tubes. To efficiently preserve the matrix on the surface of the PDMS, the Falcon tubes were connected to a freeze dryer (Labconco FreeZone 4.5) and dried at -50°C and 0.28 mTorr for 24 hours. After drying, they were gold sputter-coated and imaged using scanning electron microscopy (FEI Company Quanta 650 FEG Scanning Electron Microscope).

### TEM imaging

Ultrastructural analysis of the hiPSC-CM spheroids was done using TEM imaging. Once the spheroids were collected, they were fixed in a 2.5% glutaraldehyde solution for 1 hour at room temperature and given to the High Resolution Imaging Facility for further processing. Prepared sample blocks were completely sectioned using a diamond knife for clean and even sections. Samples were mounted and viewed using the Tecnai Spirit T12 Transmission Electron Microscope. Images were collected for each group of spheroids.

### Optical mapping

To assess action potential duration, conduction velocity, and minimum pacing cycle length, spheroids were stained with a voltage sensitive dye RH-237 (2.5 μM) for 5 minutes, transferred to a perfusion chamber mounted on an inverted microscope, and held in place with two nylon meshes (280-μm pore size). The bottom mesh was secured in place while the top mesh was mounted on a micromanipulator and positioned to hold spheroids with minimal pressure. Spheroids were perfused with Hank's balanced salt solution at approximately 37°C. Spheroids were stimulated using a bipolar electrode consisting of a glass pipette filled with Hank’s solution and a silver wire coiled around its tip. The electrode tip was positioned at a spheroid edge using a micromanipulator. Stimulation pulses had rectangular shape with duration of 2 ms and current strength 1.5-times the excitation threshold. Fluorescence was excited with a 200-W Hg/Xe arc lamp and recorded with a 16×16 photodiode array (Hamamatsu) at a spatial resolution of 110 μm per diode as previously described [16]. The excitation light was filtered at 532–587 nm and emitted fluorescent light was filtered at > 650 nm. To eliminate motion artifacts caused by spheroid contractions, the perfusion solution was supplemented with 5 μM of blebbistatin. Activation times were measured at 50% of the maximum action potential amplitude and used to construct isochronal maps of activation spread. Conduction velocity was calculated at each recording site from local activation times and averaged across the whole mapping area. Action potential duration was measured at 50% of signal recovery (APD50). Stimulation cycle length was decreased from 1000 ms to 200 ms to assess the spheroids’ ability to activate at fast stimulation rates.

### Immunolabeling and imaging of hiPSC-CM spheroids

Spheroids were first placed in OCT compound, frozen overnight in -80°C, and subsequently cryosectioned. Immunolabeling of spheroid cryo-sections were carried out at room temperature in ambient light conditions. Sections were blocked with 3% BSA containing 10% normal donkey serum for 30 minutes followed by immunolabeling with primary antibodies (S2 Table) in blocking buffer for 45 minutes. After probing with fluorescein labeled appropriate secondary antibodies and nuclei counter stained with DAPI for 45 minutes, sections were mounted. Once completed, the sections were examined using a fluorescence microscope (Olympus IX83 Fluorescent Microscope) as well as a confocal microscope (Olympus FV3000 Confocal Microscope).

### Statistical analysis

The n represents the number of spheroids collected from a single differentiation batch. For these set of experiments, we collected spheroids from 3–4 different batches (stated where necessary). For statistical analysis, data are shown in the form mean ± SEM. Significance was chosen as p < 0.05. This was determined using both one and two way ANOVA. These analyses were performed utilizing Microsoft Excel’s data analysis software package.

## Results

### hiPSC-CM spheroid formation

**Fig 1:**
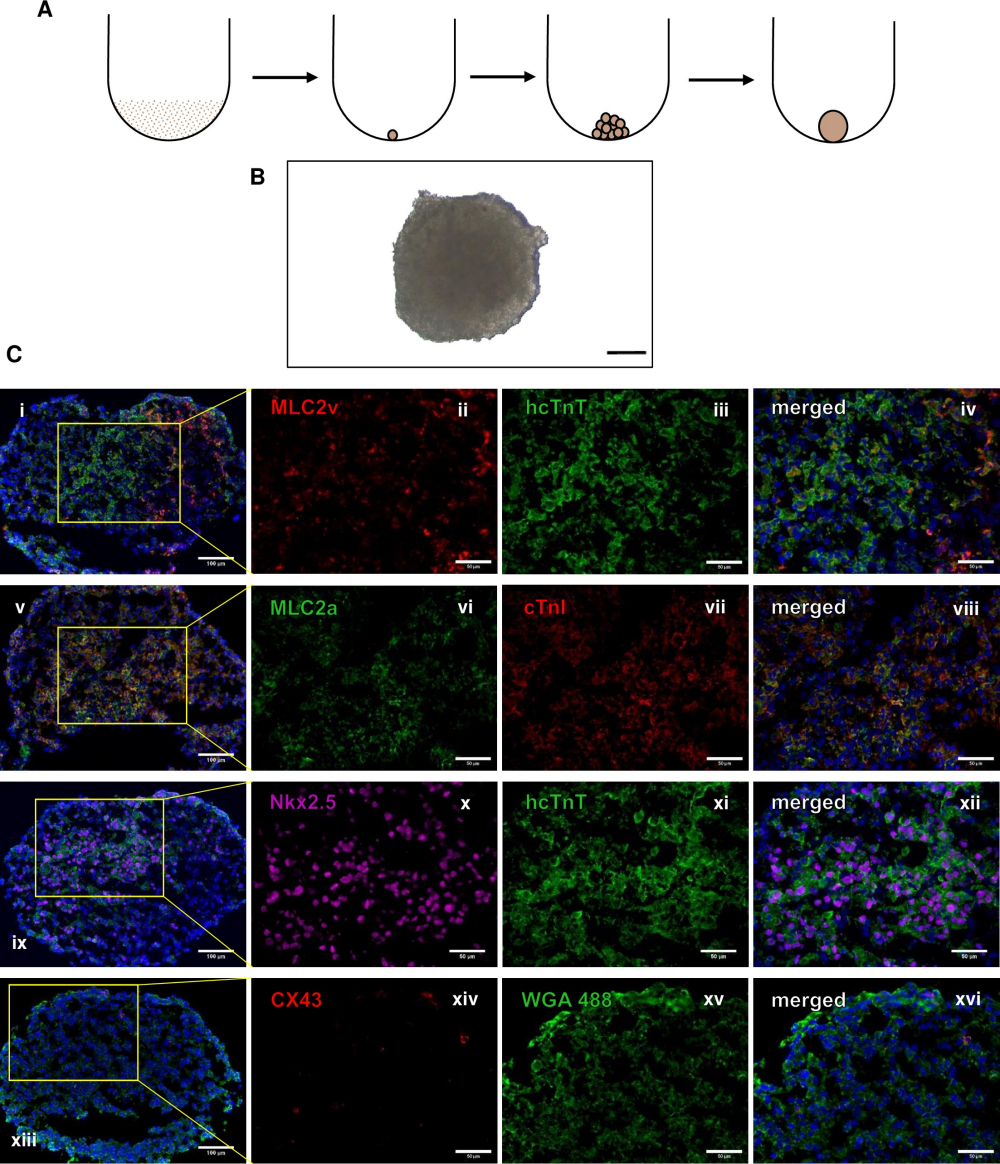
Generation and characterization of hiPSC-CM spheroids. (A) Small spheroids are generated using ultra-low retention plates with approximately 2.5x10^3^ to 3x10^5^ cells in suspension depending on the required size. They are added together to make larger spheroids that are approximately 800 μm in diameter. (B) Brightfield image of spheroid. Scale bar: 200 μm. Spheroids were immunolabeled for cardiomyocyte markers: (C) (i-iv) MLC2v and hcTnT, (v-viii) MLC2a and cTnI, (ix-xii) Nkx 2.5 and hcTnT, and (xiii-xvi) CX43 and WGA. Scale bar: (column 1) 100 μm, (columns 2–4) 50 μm.

hiPSC-CM spheroids were generated from the addition of smaller spheroids into a round bottom tube (Fig 1A and 1B) and all had a diameter of approximately 800 μm. After the spheroids were made, they were isolated and subjected to immunolabeling for cardiac specific markers Nk2 Homeobox 5 (Nkx 2.5), human cardiac troponin T (hcTnT), ventricular myosin light chain 2 (MLC2v), atrial myosin light chain 2 (MLC2a), and gap-junctional protein connexin 43 (CX43) (Fig 1C). These data confirmed the identity of the cells of the spheroids as cardiomyocytes.

### Strain achieved with cyclic stretching device and PDMS molds and effective electrical stimulation

**Fig 2:**
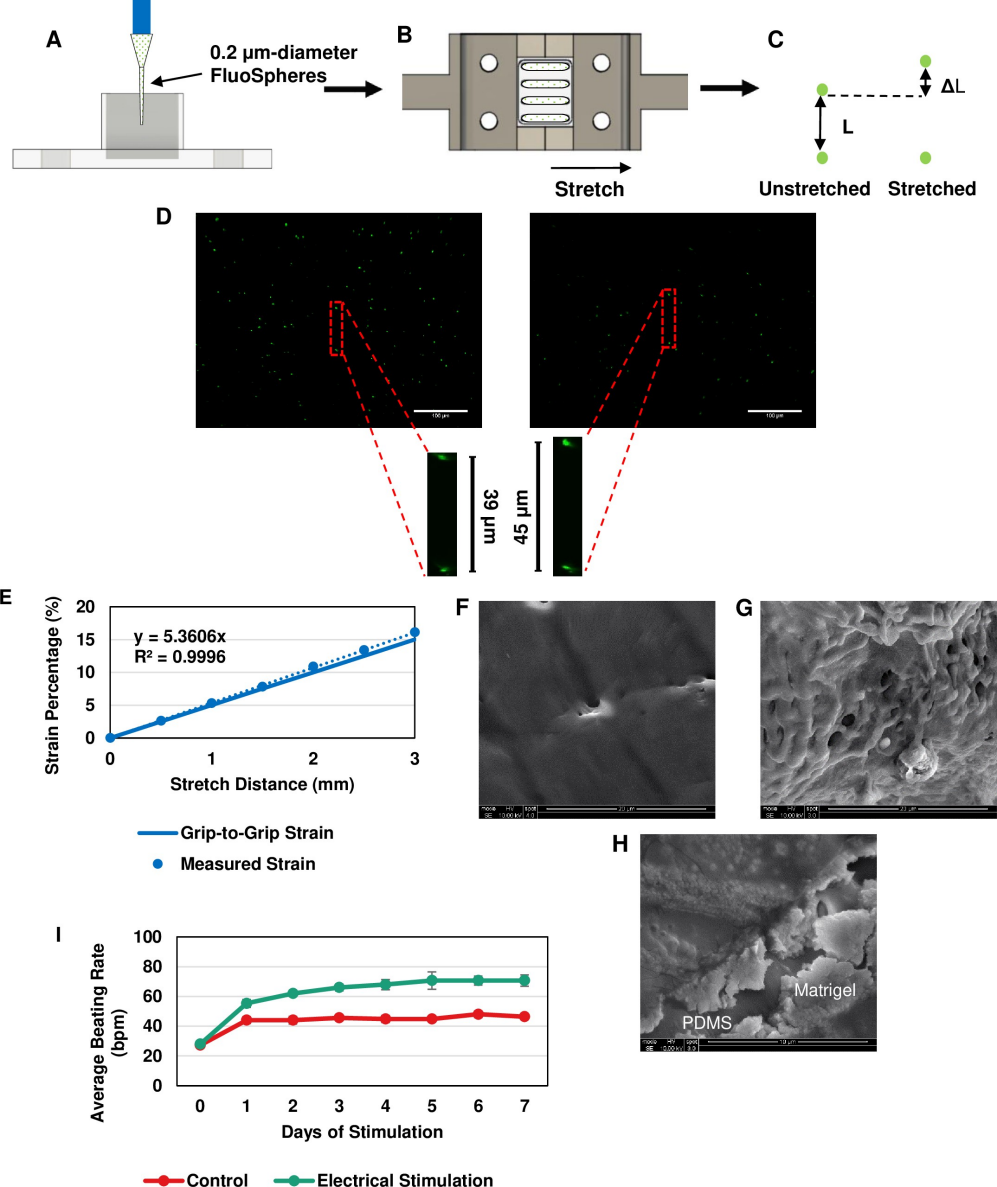
Confirmation of effectiveness of cyclic stretching and electrical stimulation setups. Fluorescent microspheres were used to measure and standardize the stretching characteristics of the PDMS mold. (A) Microspheres were loaded within each channel and allowed to settle to the bottom, (B) the mold was then attached to the stretching blocks and statically stretched to distances ranging from 0 to 3 mm (based on the actuator distance), and (C) two aligned spheres were chosen to measure the change in distance between them at each increment along with the subsequent strain. (D) Images of the fluorescent spheres at baseline and 3 mm. Scale bar: 100 μm. (E) The generated standard curve from data collected compared to the grip-to-grip strain values. SEM image of (F) only PDMS, (G) PDMS + Matrigel, and (H) combination image of PDMS surface and Matrigel coating. (I) Average beating rate measurement over 7 days of electrical stimulation. These results indicate that each of these stimulation methods were effective in providing the desired interventions of electrical and mechanical stimulation on the spheroids.

To confirm that the PDMS channels were experiencing the desired strain while being stretched by the device, fluorescent spheres (0.2 μm FluoSpheres Amine-Modified Microspheres, Thermo Fisher Scientific, Product #F8764) in PBS were added to the channels and allowed to settle in the bottom overnight. Once settled the channels were stretched in the device at lengths between 0.5 mm and 3 mm (Fig 2A and 2B). Two centrally located spheres were chosen that were in a vertically aligned relationship and the distance between the two was measured at each length increment. To calculate the strain, the change in distance between the spheres was divided by the original distance and converted to a percentage (Fig 2C). For each preparation, three separate groups were chosen for measurements (Fig 2D). A standard curve was generated from the data and compared to the grip-to-grip strain, which was used as an estimate for applying stretch (Fig 2E). Measured strains higher than 10% exhibited larger deviation from the grip-to-grip strain. However, because the largest strain used in this experiment was 10%, the grip-to-grip measurement was a good approximation for our experiments.

In addition, Matrigel was used in these experiments to coat the surface of the PDMS for spheroid attachment so determining whether Matrigel was present on the PDMS surface was critical to the assumption that they were attached during stretching. To do this, SEM was utilized. From the images collected, the PDMS presented a smooth, flat surface (Fig 2F), while the Matrigel coated surface showed a globular, protein structure (Fig 2G). From this, it was determined that Matrigel could efficiently attach to the surface of the PDMS channels allowing for the subsequent attachment of the spheroids.

To test the effectiveness of the electrical culture-pacing setup, single spheroids (n = 5 control and n = 6 stimulated) were stimulated at 6.5 V/cm at 5 ms pulse duration with a frequency of 2 Hz, as determined through threshold measurements. The spheroids were stimulated for 7 days and the beating rate of each spheroid during stimulation was measured every 24 hours and compared to unstimulated spheroids (Fig 2I). The average beating rate of the stimulated spheroids increased from 28.0±1.5 bpm at Day 0 to 70.7±3.8 bpm at Day 7, whereas the unstimulated spheroids increased from 27.2±1.5 bpm at Day 0 to 46.4±1.0 bpm at Day 7. The beating rate adaptation of hiPSC-CM has been well documented [17]. Therefore based on these data, the spheroids were being effectively stimulated using this monolayer culture-pacing setup.

### Chronic, electrical stimulation increases cardiac gap junction and contractile machinery transcript and protein expression

**Fig 3:**
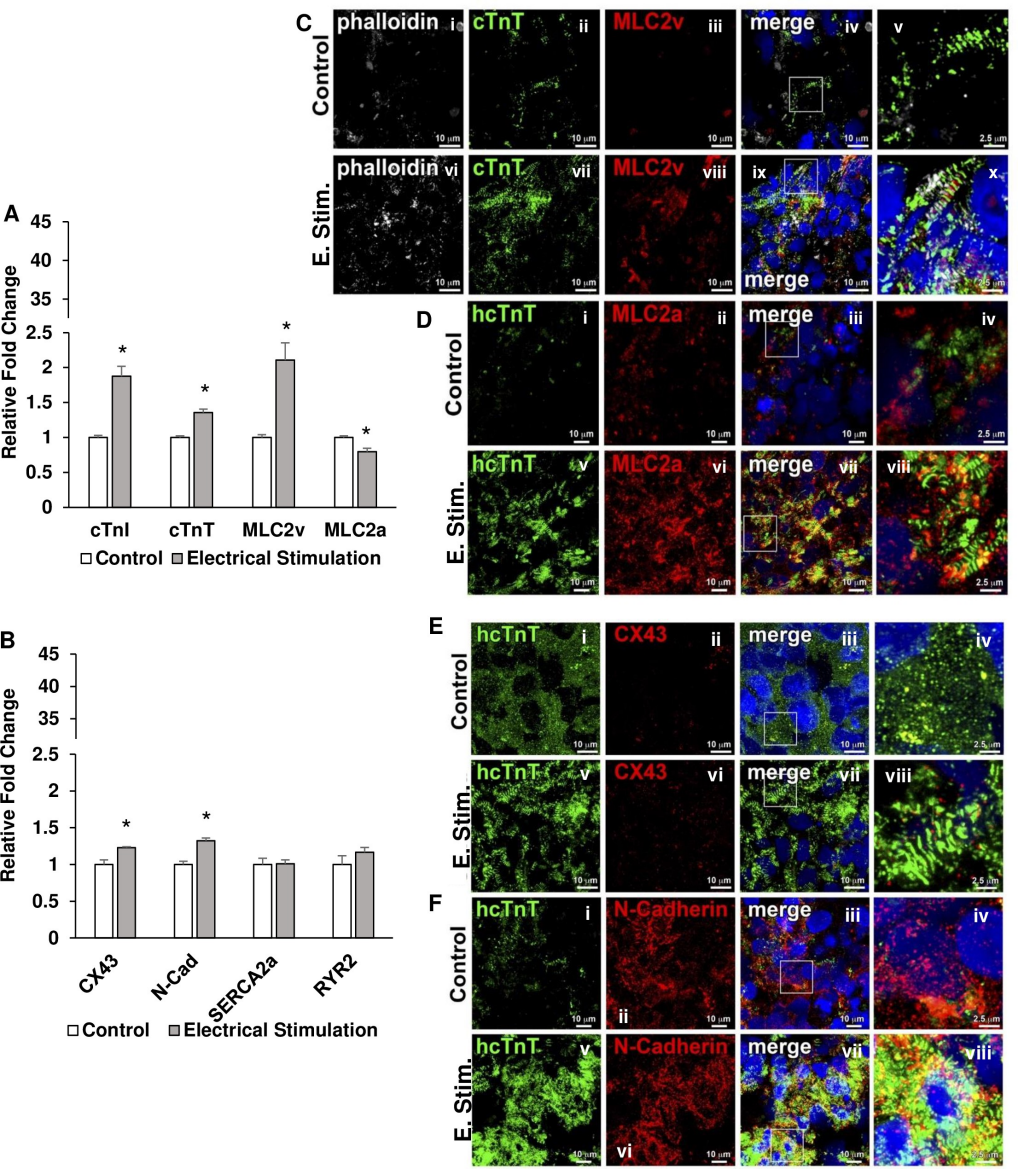
RT-qPCR analysis and immunolabeling of stimulated spheroids. hiPSC-cardiomyocyte spheroids were collected after 7 days of electrical stimulation. Transcript expression was analyzed using RT-qPCR. Control vs Electrical Stimulation: (A) cTnI, cTnT, MLC2v, and MLC2a; (B) CX43, N-Cad, SERCA2a, and RYR2. n = 6 for both groups, * p<0.05. In addition, spheroid cross sections from both control and electrical stimulation groups were immunolabeled for cardiomyocyte maturation markers as follows: (C) (i-x) phalloidin, cTnT, and MLC2v; (D) (i-viii) hcTnT and MLC2a; (E) (i-viii) hcTnT and CX43; (F) hcTnT and N-Cad. Scale bar: (C(i-iv, vi-ix), D(i-iii, v-vii), E(i-iii, v-vii), F(i-iii, v-vii)) 10 μm and (C(v, x), D(iv, viii), E(iv, viii), F(iv, viii)) 2.5 μm.These results indicate spheroids exposed to electrical stimulation developed improved transcript expression of gap-junctional and ventricular cardiomyocyte proteins. In addition, immunolabeling revealed an improved striated muscle formation, induced MLC2v expression, and increased cell-cell communication potential with the improvement of expression of CX43 and N-Cad.

The spheroids were exposed to electrical stimulation using a monolayer culture-pacing system. After being added to a well with media, the stimulating electrodes were submerged into the media. Pacing was carried out with square wave pulses at 6.5 V/cm with 5 ms pulse durations at a frequency of 2 Hz for 7 days. These values were determined by adjusting the electric field strength as well as pulse duration until the spheroids reached threshold and captured the stimulation frequency. At the end of the experiment, the spheroids (n = 6 for control and stimulated groups; 3 differentiation batches) were collected for analysis. RT-qPCR data (3 replicates for each primer) revealed the changes in transcript expression of cardiac markers for maturation. The data collected indicated that CX43, N-Cad, MLC2v, cTnT, and cTnI transcript expression were increased and MLC2a transcript expression was decreased in stimulated spheroids compared to unstimulated spheroids (Fig 3A and 3B). In addition, through immunostaining, it can be seen that there was an increase in the expression of gap-junctional proteins (CX43 and N-Cad), MLC2v, and cTnT (Fig 3C–3F).

### Cyclic, uniaxial stretching increases the ratio of beta myosin heavy chain to (βMHC) to alpha myosin heavy chain (αMHC) as well as the expression of MLC2v and calsequestrin-2 (CASQ2)

**Fig 4:**
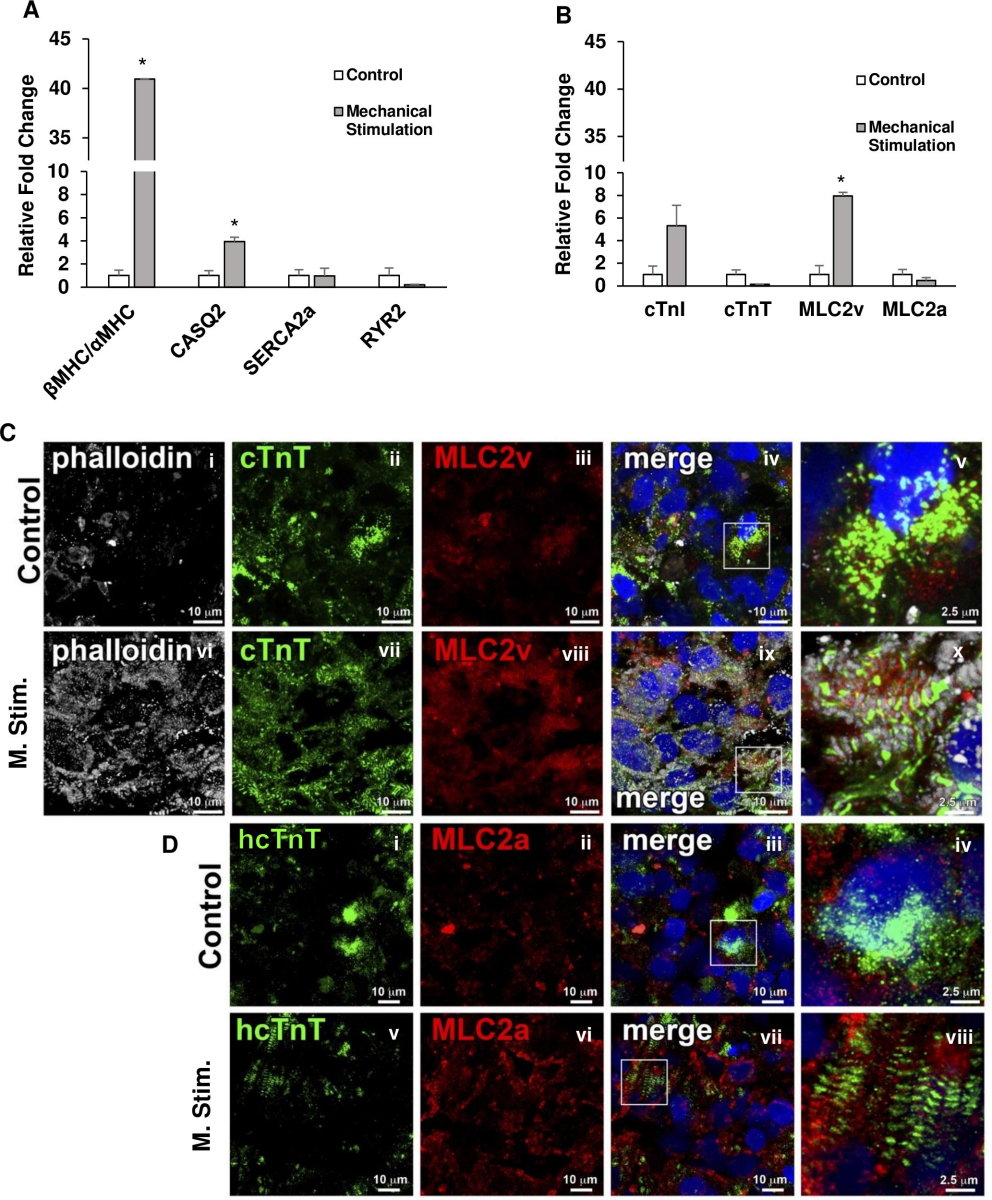
RT-qPCR and immunolabeling of mechanically stimulated spheroids. Spheroids were mechanically stimulated using the cyclic stretching device detailed previously on the custom PDMS molds at a frequency of 1 Hz with a strain of 10%. After 7 days, the spheroids were collected and analyzed. Control vs Mechanical Stimulation: (A) βMHC/αMHC, CASQ2, SERCA2a, and RYR2; (B) cTnI, cTnT, MLC2v, and MLC2a. n = 6 for both groups, * p<0.05. Control and mechanically stimulated spheroids were immunolabeled for (C) (i-x) phalloidin, MLC2v, and cTnT; (D) (i-viii) hcTnT and MLC2a. Scale bar: (C(i-iv, vi-ix) and D(i-iii, v-vii)) 10 μm and (C(v, x) and D(iv, viii)) 2.5 μm. Spheroids subjected to mechanical stimulation exhibited an increase in βMHC/αMHC transcript ratio and MLC2v and CASQ2 transcripts and this coupled with upregulation of MLC2v protein in the cardiomyocytes as seen from the immunolabeling show an enhanced ventricular cardiomyocyte formation compared to control.

The hiPSC-CM spheroids were exposed to cyclic, uniaxial stretch as described in the Methods section. Spheroids were seeded on to the Matrigel coated PDMS channel and cyclic stretching was done at 10% strain and a frequency of 1 Hz for 7 days (n = 6 for control and stretched groups; 3 differentiation batches). At the end of the experiment, the spheroids were collected for further analysis. RT-qPCR (3 replicates for each primer) was done to quantify the transcript expression levels of cardiac specific markers such as cTnI, cTnT, MLC2v, MLC2a, βMHC, αMHC, CASQ2, SERCA2a, and RYR2. Interestingly, the ratio of βMHC to αMHC transcript expression was found to be significantly increased in the stretched spheroids. Additionally, MLC2v and CASQ2 transcript expression were also found to be increased in the stretched spheroids compared to the unstretched group (Fig 4A and 4B). Immunolableing of MLC2v and MLC2a were performed to confirm the structural maturation of spheroids (Fig 4C and 4D). MLC2v, ventricular cardiomyocyte marker, was observed to be upregulated upon mechanical stimulation, while no changes were seen in MLC2a expression level.

### Chronic, electrical stimulation and cyclic, uniaxial stretching improved ultrastructural development of hiPSC-CM spheroids

**Fig 5:**
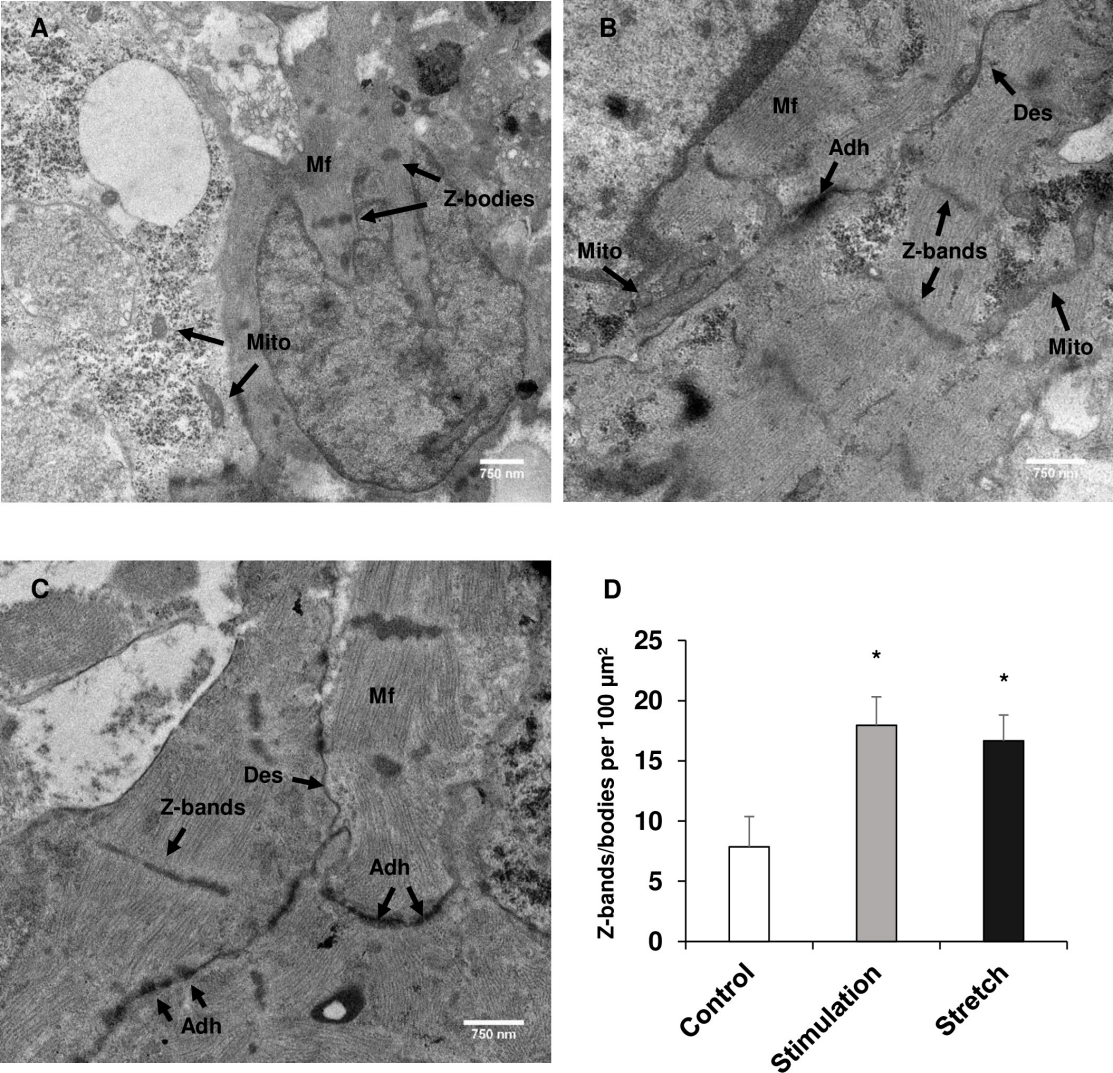
Ultrastructural maturation of hiPSC-CM spheroids. Ultrastructural maturation of hiPSC-cardiomyocyte was assessed using TEM after 7 days of normal culture, electrical stimulation, and mechanical stimulation. (A) Control, (B) electrical stimulation, and (C) mechanical stimulation. “Mito”: mitochondria; “Mf”: myofibers; “Des”: desmosome; “Adh”: adherens junction. Scale bar: 750 nm. (D) Quantification of the number of Z-bands/bodies per 100 μm^2^. n = 3–5. * p<0.05. Both stimulation regimens enhanced the ultrastructure of the cardiomyocytes with electrical stimulation significantly improving z-line formation as well as fiber orientation and mitochondrial location, while mechanical stimulation also significantly improved z-line formation as well as adherens junction expression compared to the control group.

After the spheroids were electrically (n = 4) and mechanically stimulated (n = 5), they were imaged using TEM to elucidate whether the ultrastructure of the hiPSC-CM spheroids had been improved. The control spheroids (Fig 5A) exhibited limited number of Z-bands indicating these cardiomyocytes are of neonatal phenotype. Interestingly, Z-band formation and structure was found to be improved in the electrically stimulated group (Fig 5B). Additionally, features like mitochondrial arrangement around the contractile fibers and junctions between cardiomyocytes were prominent in the electrical stimulated group. The mechanically stretched group (Fig 5C), while the Z-bands were not as structurally formed as the electrically stimulated group, formed many more Z-bodies, which are precursors to Z-bands, adjacent contractile fibers, and junctions when compared to the control group of spheroids (n = 3). The number of Z-bands and groups of Z-bodies formed per area (100 μm^2^) was quantified and was found to be significantly increased in both the stimulated and stretched groups compared to the control group of spheroids (Fig 5D).

### Electrical stimulation enhanced the spheroid ability to activate at fast rates

**Fig 6:**
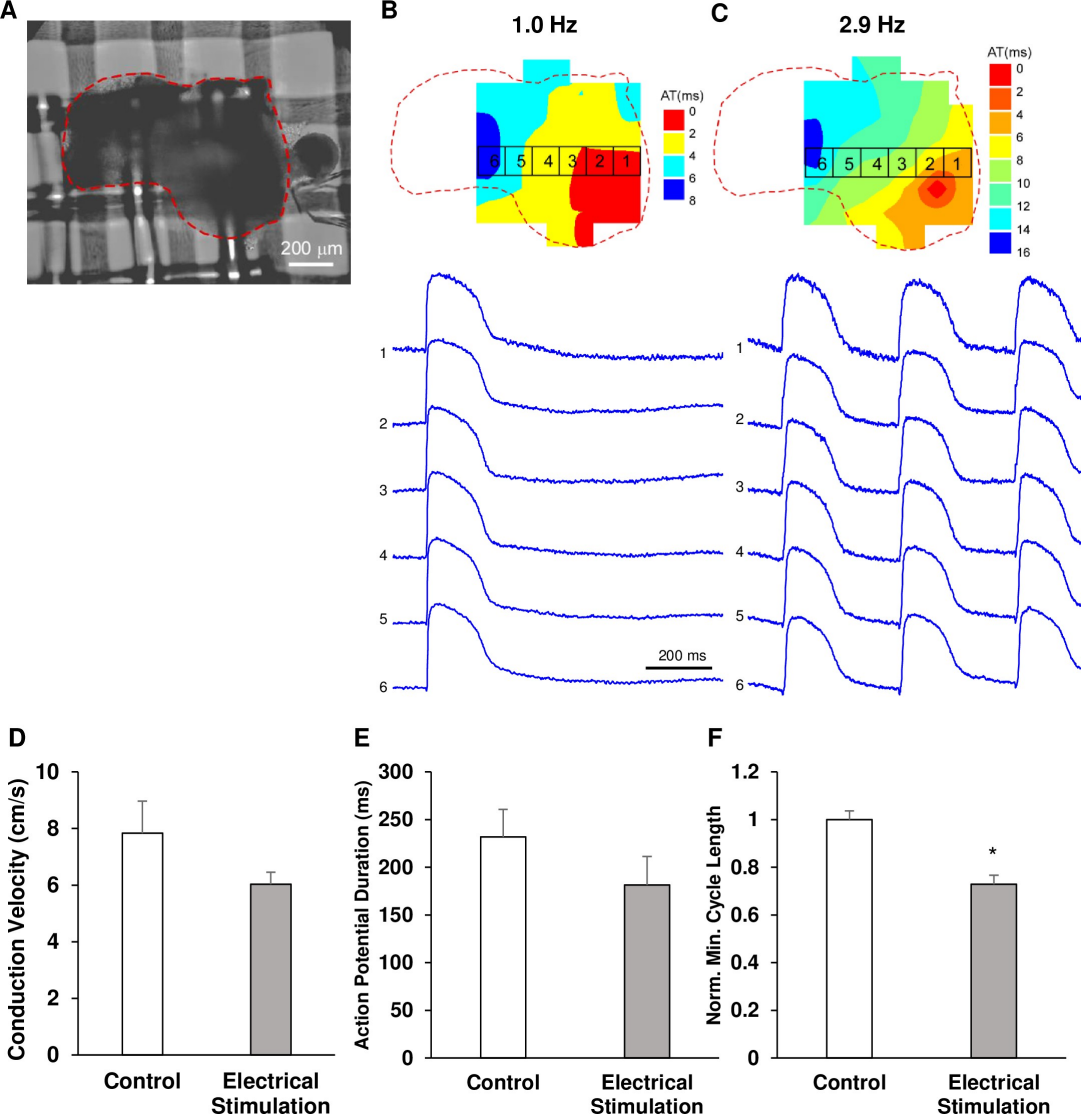
Optical mapping of hiPSC-CM spheroids. Isochronal maps and corresponding action potentials in an electrically stimulated spheroid (A) at a cycle length of 1000 ms (B) and the minimum cycle length of 350 ms (C). (D) Average conduction velocity in control and electrically stimulated spheroids (n = 5 for both). (E) Average APD50 in control and electrically stimulated groups (n = 5 for both). (F) Average normalized minimum cycle length in control and electrically stimulated groups (n = 8 for both). * p<0.05. Although there was no statistically significant difference between the spheroids as it pertains to conduction velocity and APD50, spheroids exposed to electrical stimulation were able to synchronize their beating and generate action potentials at higher frequencies of pacing compared to control spheroids.

The ability of spheroids to generate action potentials was examined by optical mapping of V_m_ during pacing with cycle length varying between 1000 ms (1 Hz) to 200 ms (5 Hz). Fig 6 shows an example of activation spread and action potential recordings in a spheroid from the electrically stimulated group (Fig 6A) at a cycle length of 1000 ms (Fig 6B) and the minimal cycle length of 350 ms (Fig 6C). Comparison of all control (n = 5 from 4 differentiation batches) and stimulated (n = 5 from 4 differentiation batches) spheroids revealed that action potential duration measured during pacing with long CL was not significantly different in two groups. APD50 in control and electrically stimulated spheroids was 217±69 and 181±67 ms, respectively (Fig 6E). Conduction velocity was also not significantly different in two groups (7.8±2.5 vs 6.0±0.9 cm/s, respectively, Fig 6D). However, it was found that electrically stimulated spheroids were able to beat at faster pacing rates than control spheroids. The stimulated spheroids (n = 8 from 4 differentiation batches) generated action potentials at a minimum pacing cycle length of 338±21 ms (~2.96 Hz), while unstimulated spheroids (n = 8 from 4 differentiation batches) could be activated only at a minimum pacing cycle length of 569±92 ms (~1.76 Hz). Because of the significant variability in the minimum pacing CL between different batches of spheroids, absolute measurements did not show statistical significance. However, inside each batch the minimum CL in stimulated spheroids was consistently smaller than in the control spheroids and the minimum CL values normalized by the control batch average value were significantly different (Fig 6F).

## Discussion

Cellular spheroids are seen as tremendous potential in drug screening [18, 19] and disease modeling for their small size and three dimensional nature. Given the various sizes these cellular constructs can be, they can be cultured in clinically-relevant amounts via multiple methods at a quick rate. However, when generated from hiPSC-CMs, they still do not adequately possess the hallmark features of mature, adult cardiomyocytes *in vivo* which affects their reliability in drug discovery and disease modeling [14, 19]. Improving the maturity of these cells is paramount for these constructs to be adequate tissue surrogates for these endeavors, but it is relatively unknown as to how they will respond to maturation regimens such as electrical and mechanical stimulation.

Using the scaffold-free, three-dimensional hiPSC-CM spheroids and the cyclic stretch and electrical stimulation, the main findings of the present study are: 1) enhanced maturity of hiPSC-CMs on various levels as evidenced by the enhanced gap junction coupling through the major ventricular gap junctions CX43 and N-Cad, increased expression of MLC2v in both the electrical and mechanical stimulation groups as well as the increase in the ratio of βMHC and αMHC, and enhanced ability of the spheroids exposed to electrical stimulation to capture higher rates of pacing; 2) improved ultrastructure of the cardiomyocytes with improved contractile fiber arrangement and number, mitochondrial location, and Z-band/body number in each group of spheroids compared to the control group; and 3) confirmed the reliability of the custom cyclic, uniaxial stretching device to effectively stretch the PDMS substrate along with the spheroids attached to the surface as well as the reliability of the electrical stimulation system to stimulate the spheroids.

Adult cardiomyocytes are arranged and connected in such a way for the most efficient propagation of signal in the form of gap junctions. However, hiPSC-CMs do not possess an abundance of these necessary connections nor the proper arrangement. As in a similar study using electrical stimulation on modified hiPSC-CM spheroids [20], we found there was an increase in gap junction expression within the spheroids. CX43 was upregulated in the spheroids along with N-Cad showing that the cardiomyocytes within the spheroids were creating improved connections both mechanically and electrically. However, unlike the previous study, no artificial materials were added to these spheroids to help with signal propagation and gap junction expression. The spheroid model which we present here is a better representation of the *in vivo* environment when compared to models that incorporate conductive nanomaterials.

Another sign of the improved maturation was the increase in expression of MLC2v and the decrease in expression of MLC2a. Within the heart, cardiomyocytes of the atria uniquely express MLC2a while those of the ventricles uniquely express MLC2v [21]. Generating cardiomyocytes that are more ventricle-like in nature could prove beneficial for future cell therapy experiments involving the ventricles of the heart as well as disease modeling and drug discovery as this would allow for a more homogeneous population of cells. Richards et al. showed that the addition of conductive silicon nanowires into hiPSC-CM spheroids could improve the expression of MLC2v both on the transcript and protein levels after electrical stimulation. However, they showed no change in the MLC2a expression on either level. We were able to not only enhance the expression of MLC2v, but we were able to decrease the expression of MLC2a without the addition of conductive nanomaterials. This finding points to an improved ventricular phenotype of the cardiomyocytes within the spheroids.

Also, cTnI expression is another marker of improved maturation [22, 23]. The expression of cTnI increased in both stretched and stimulated groups pointing towards more adult-like hiPSC-CMs. Additionally, in the mechanical stimulation group, the ratio of βMHC and αMHC significantly increased when compared to the control group. This ratio increase suggests an improved hiPSC-CM maturity through enhanced contractile machinery [24].

Ultrastructural analysis was done on the spheroids after each maturation regimen and revealed that contractile fiber length and organization was improved with the increased number of Z-bands and Z-bodies formed. The formation of alpha actinin-rich Z-bodies is an early step in myofibrillogenesis in the developing heart [25]. The electrically stimulated spheroids had a greater number of Z-bands that formed from Z-bodies when compared to the unstimulated group, possessing improved fibril orientation and structure. In addition, the mechanically stretched spheroids had an increased amount of Z-bodies as well as an increased number of fibers oriented around the Z-bodies beginning fibril formation. This improvement in myofibrillogenesis in both groups also demonstrates an enhanced maturation of hiPSC-CMs similar to what was recently reported [26]. Ronaldson-Bouchard et al. showed significant improvements in the ultrastructure of hiPSC-CMs with increased mitochondrial density around the contractile machinery and improved number of band structures after electrical stimulation of an engineered tissue. Through our experiments, we were able to show comparable improvements in an intact spheroid construct.

Functional maturation was also assessed using optical mapping which revealed that electrically stimulated spheroids possessed the ability to depolarize and repolarize efficiently at a significantly decreased cycle lengths during pacing compared to unstimulated spheroids (p<0.05, 338±21 ms vs. 569±92 ms; ~2.96 Hz vs. ~1.76 Hz). This improvement in cell electrophysiology coupled with improved structural maturity, suggest a decreased arrhythmogenic potential if used *in vivo* as a cellular therapeutic intervention. hiPSC-CMs and their use in cell therapy in the heart has always been plagued by their potential to cause arrhythmias [27]. While we do not have *in vivo* data to support this claim, the ability of the electrically stimulated spheroids to respond to increased rates of pacing *in vitro* shows that they do possess the enhanced machinery to handle varying rates of beating that would occur in an animal model.

In conclusion, we were able to achieve an enhanced structural maturity of hiPSC-CM spheroids as well as an improved electric pacing handling ability compared to control spheroids. Exposing hiPSC-CM spheroids to electrical stimulation and mechanical stretch promote maturation as evidenced by the increase expression of gap junction and calcium handling machinery at the transcript, protein, and ultrastructural levels. Thus, the use of electrical and mechanical stimuli on scaffold-free hiPSC-CM spheroids enhanced the maturation on multiple levels which could potentially lead to better surrogates for drug testing, disease modeling, or therapeutic approaches.

## Supporting information

S1 Fig Methods for providing cyclic, uniaxial stretch and chronic, electrical stimulation to spheroids. (A) The PDMS molds have four channels that are each 1 mm wide by 8.5 mm long with a depth of 9 mm to allow for the spheroids to effectively settle on the bottom to attach.(B) Representative images of the mold with empty channels and with spheroids added.(C) After the spheroids are added to the channels, they are attached to metal stretching blocks where one end is moved by cyclic actuators and the other is kept motionless.(D) For electrical stimulation, the spheroids are added in a dish between two electrodes which are then activated to generate an electric field thereby stimulating the spheroids.
(PDF)

S1 Table RT-qPCR primers used in this study. Primers were acquired from Invitrogen and used to detect the genetic changes in cardiomyocyte maturation markers after electrical and mechanical stimulation.
(PDF)

S2 Table Primary antibodies used in this study. Antibodies used to characterize the hiPSC-CM spheroids before and after both stimulation regimens to analyze the change in expression of cardiomyocyte maturation proteins.
(PDF)

S1 Dataset Data supplement for figures. (XLSX)
