# Correction: Maturation of three-dimensional, hiPSC-derived cardiomyocyte spheroids utilizing cyclic, uniaxial stretch and electrical stimulation

**DOI:** 10.1371/journal.pone.0223424

**Published:** 2019-09-30

**Authors:** Wesley LaBarge, Saidulu Mattapally, Ramaswamy Kannappan, Vladimir G. Fast, Daniëlle Pretorius, Joel L. Berry, Jianyi Zhang

The second author’s name is spelled incorrectly. The correct name is: Saidulu Mattapally. The correct citation is: LaBarge W, Mattapally S, Kannappan R, Fast VG, Pretorius D, Berry JL, et al. (2019) Maturation of three-dimensional, hiPSC-derived cardiomyocyte spheroids utilizing cyclic, uniaxial stretch and electrical stimulation. PLoS ONE 14(7): e0219442. https://doi.org/10.1371/journal.pone.0219442
