# Supplementary material for: Maturation of three-dimensional, hiPSC-derived cardiomyocyte spheroids utilizing cyclic, uniaxial stretch and electrical stimulation
Source: PLoS One. 2019 Jul 5;14(7):e0219442. doi: 10.1371/journal.pone.0219442 (PMC6611624; doi:10.1371/journal.pone.0219442)
Supplement: S1 Fig — (A) The PDMS molds have four channels that are each 1 mm wide by 8.5 mm long with a depth of 9 mm to allow for the spheroids to effectively settle on the bottom to attach.(B) Representative images of the mold with empty channels and with spheroids added.(C) After the spheroids are added to the channels, they are attached to metal stretching blocks where one end is moved by cyclic actuators and the other is kept motionless.(D) For electrical stimulation, the spheroids are added in a dish between two electrodes which are then activated to generate an electric field thereby stimulating the spheroids. (PDF) [file pone.0219442.s001.pdf]

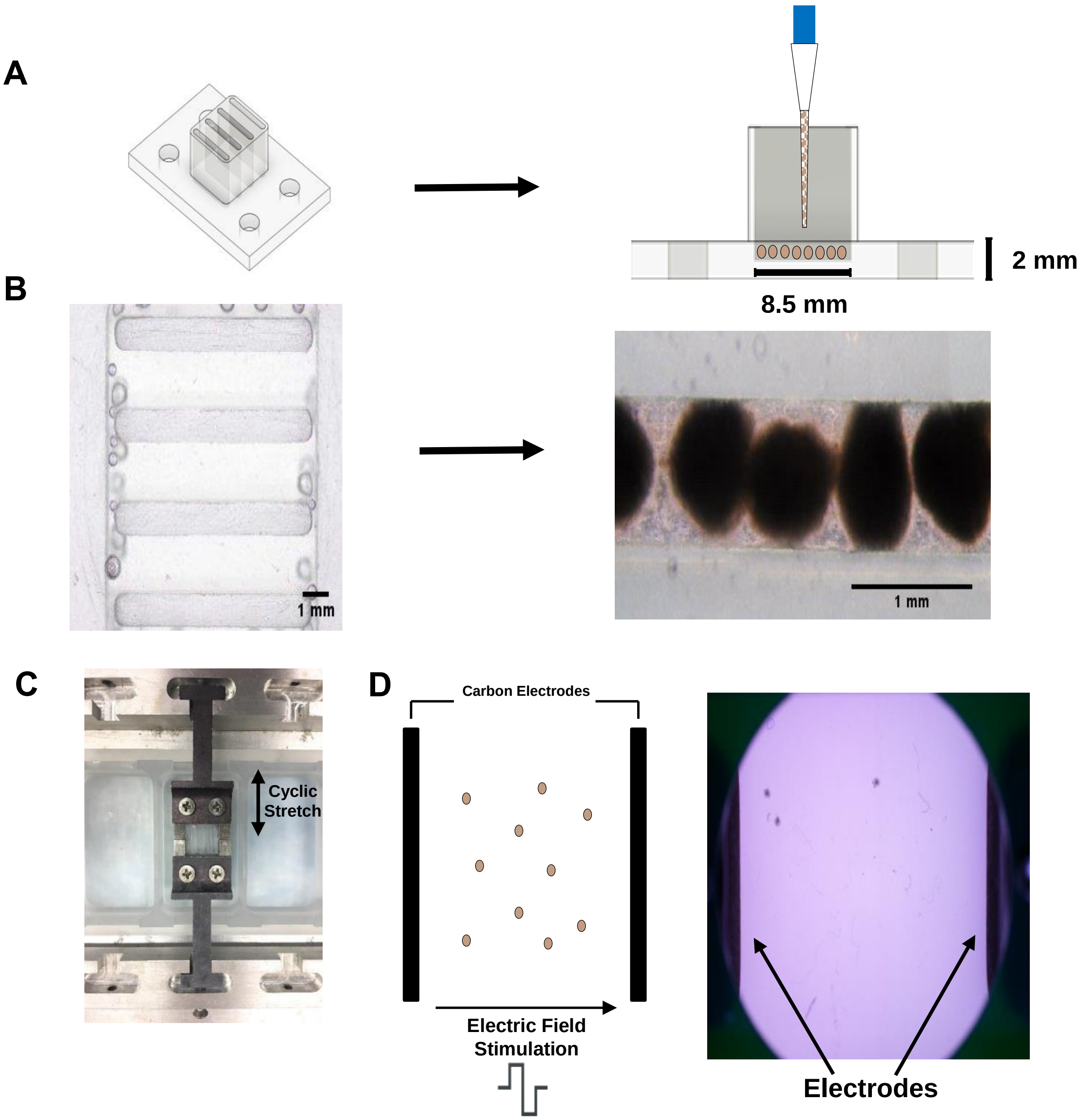

S1 Fig. Methods for providing cyclic, uniaxial stretch and chronic, electrical stimulation to spheroids. (A) The PDMS molds have four channels that are each 1 mm wide by 8.5 mm long with a depth of 9 mm to allow for the spheroids to effectively settle on the bottom to attach. **(B)** Representative images of the mold with empty channels and with spheroids added. **(C)** After the spheroids are added to the channels, they are attached to metal stretching blocks where one end is moved by cyclic actuators and the other is kept motionless. **(D)** For electrical stimulation, the spheroids are added in a dish between two electrodes which are then activated to generate an electric field thereby stimulating the spheroids.
