# Supplementary material for: Maturation of three-dimensional, hiPSC-derived cardiomyocyte spheroids utilizing cyclic, uniaxial stretch and electrical stimulation
Source: PLoS One. 2019 Jul 5;14(7):e0219442. doi: 10.1371/journal.pone.0219442 (PMC6611624; doi:10.1371/journal.pone.0219442)
Supplement: S1 Table — Primers were acquired from Invitrogen and used to detect the genetic changes in cardiomyocyte maturation markers after electrical and mechanical stimulation. (PDF) [file pone.0219442.s002.pdf]

Genes	*Gene Product*	*Forward Primer*	*Reverse Primer*	*Product Length (bp)*	*Annealing Temperature (°C)*	*GenBank Accession No.*
GJA1	CX43	5'-GGTCTGAGTGCCTGAACTTGCCT-3'	5'-AGCCACACCTTCCCTCCAGCA-3'	184	60	NM_000165.4
CDH2	N-Cad	5'-AGCCAACCTTAACTGAGGAGT-3'	5'-GGCAAGTTGATTGGAGGGATG-3'	136	60	NM_001792.4
ATP2A2	SERCA2a	5'-TCACCTGTGAGAATTGACTGG-3'	5'-AGAAAGAGTGTGCAGCGGAT-3'	149	60	NM_001681.3
RYR2	RYR2	5'-TTGGAAGTGGACTCCAAGAAA-3'	5'-CGAAGACGAGATCCAGTTCC-3'	141	60	NM_001035.2
TNNT2	cTnT	5'-TTCACCAAAGATCTGCTCCTCGCT-3'	5'-TTATTACTGGTGTGGAGTGGGTGTGG-3'	166	60	NM_001276346.1
TNNI3	cTnI	5'-CCTCACTGACCCTCCAAACG-3'	5'-GAGGTTCCCTAGCCGCATC-3'	104	60	NM_000363.4
MYL2	MLC2v	5'-ACATCATCACCCACGGAGAAGAGA-3'	5'-ATTGGAACATGGCCTCTGGATGGA-3'	247	60	NM_000432.3
MYL7	MLC2a	5'-GGAGTTCAAAGAAGCCTTCAGC-3'	5'-AAAGAGCGTGAGGAAGACGG-3'	178	60	NM_021223.2
CASQ2	CASQ2	5'-GTTGCCCGGGACAATACTGA-3'	5'-CTGTGACATTCACCACCCCA-3'	142	60	NM_001232.3
GAPDH	GAPDH	5'-TCGACAGTCAGCCGCATCTTCTTT-3'	5'-ACCAAATCCGTTGACTCCGACCTT-3'	94	60	NM_002046.6

S1 Table. RT-qPCR primers used in this study. Primers were acquired from Invitrogen and used to detect the genetic changes in cardiomyocyte maturation markers after electrical and mechanical stimulation.
