# Supplementary material for: Maturation of three-dimensional, hiPSC-derived cardiomyocyte spheroids utilizing cyclic, uniaxial stretch and electrical stimulation
Source: PLoS One. 2019 Jul 5;14(7):e0219442. doi: 10.1371/journal.pone.0219442 (PMC6611624; doi:10.1371/journal.pone.0219442)
Supplement: S2 Table — Antibodies used to characterize the hiPSC-CM spheroids before and after both stimulation regimens to analyze the change in expression of cardiomyocyte maturation proteins. (PDF) [file pone.0219442.s003.pdf]

**Primary antibodies**	**Dilutions**	**Source**	**Cell target**	**Catalog #**	**Type**
**Cardiac myocyte markers**					
Human Cardiac Troponin T (hcTnT)	1:200	Abcam	Human cardiomyocyte	ab91605	Rabbit monoclonal
Cardiac Troponin I (cTnI)	1:200	Abcam	Cardiomyocyte	ab47003	Rabbit polyclonal
Ventricular Myosin Light Chain 2 (MLC2v)	1:100	Abcam	Ventricular cardiomyocyte	ab92721	Rabbit monoclonal
Atrial Myosin Light Chain 2 (MLC2a)	1:100	Abcam	Atrial cardiomyocyte	ab68086	Mouse polyclonal
Connexin 43 (CX43)	1:100	Abcam	Cardiomyocyte	ab11370	Rabbit polyclonal
N-Cadherin (N-Cad)	1:100	Abcam	Cardiomyocyte	ab18203	Rabbit polyclonal
Nkx 2.5	1:100	Thermo Fisher	Cardiomyocyte	PA5-47322	Goat polyclonal

S2 Table: Primary antibodies used in this study. Antibodies used to characterize the hiPSC-cardiomyocyte spheroids before and after both stimulation regimens to analyze the change in expression of cardiomyocyte maturation proteins.
